# MECHANISMS IN ENDOCRINOLOGY: The pathophysiology of transient congenital hypothyroidism

**DOI:** 10.1530/EJE-21-1278

**Published:** 2022-05-19

**Authors:** Catherine Peters, Nadia Schoenmakers

**Affiliations:** 1 Department of Endocrinology, Great Ormond Street Hospital for Children, London, UK; 2 University of Cambridge Metabolic Research Laboratories, Wellcome Trust-MRC Institute of Metabolic Science, University of Cambridge, Cambridge, UK

## Abstract

Transient congenital hypothyroidism (TCH) refers to congenital hypothyroidism which spontaneously resolves in the first few months or years of life. Currently, there is a paucity of reliable markers predicting TCH at diagnosis, and the diagnosis is established following the withdrawal of levothyroxine therapy around 3 years of age. The incidence of TCH is increasing, and it is a major contributor to the overall increase in the incidence of CH in recent studies. Both genetic factors, in particular mutations affecting *DUOX2* and *DUOXA2*, and environmental factors, for example, iodine deficiency and excess, anti- TSHR antibodies and exposure to antithyroid or iodine-rich medications, may cause TCH. Resolution of TCH in childhood may reflect both normal thyroid physiology (decreased thyroid hormone biosynthesis requirements after the neonatal period) and clearance or cessation of environmental precipitants. The relative contributions and interactions of genetic and environmental factors to TCH, and the extent to which TCH may be prevented, require evaluation in future population-based studies.

## Invited Author’s profile


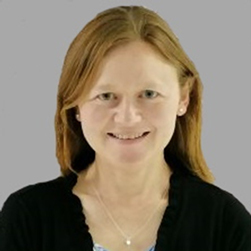


**Nadia Schoenmakers, PhD** is a Principal Investigator and Wellcome Trust Senior Clinical Fellow at NIHR Cambridge Biomedical Research Centre, University of Cambridge, UK. Dr Schoenmakers’ principal research interest lies in elucidating the genetic and environmental determinants of congenital hypothyroidism (CH). Her research uses candidate genes, gene panel and whole exome sequencing technologies to identify known and novel genetic causes of CH. She then undertakes phenotyping of genetically ascertained individuals with parallel molecular and murine studies, aiming to gain new insights into thyroid biology and associated extra-thyroidal phenotypes.

## Congenital hypothyroidism

Primary congenital hypothyroidism (CH) is the most common neonatal endocrine disorder and may be sub-classified as a failure of normal gland development (thyroid dysgenesis, TD) or failure of thyroid hormonogenesis despite the presence of a normally located thyroid gland *in situ* (GIS CH). Individuals with GIS CH may have a specific disruption of the thyroid hormone molecular biosynthetic pathway (dyshormonogenesis), which is usually genetically mediated and may result in goitre. Primary CH usually results in permanent thyroid dysfunction. However, a sizeable number of affected children will recover endogenous thyroid function in early childhood, permitting the cessation of levothyroxine treatment by the end of the third year of life. This group of children are said to have ‘transient congenital hypothyroidism’ (TCH) and usually exhibit GIS CH. Although some cases of mild dysgenesis and thyroid ectopia may have residual thyroid function, it is uncommon to see cases of TCH in this context (1, 2).

This review will clarify the definition and diagnosis of TCH and summarize key knowledge regarding its epidemiology. The process of thyroid hormone biosynthesis will be explained including the roles of known genetic and environmental determinants of TCH in normal thyroid physiology, and the likely contribution of these factors to the pathogenesis of TCH, when perturbed. Despite the prognostic importance of TCH and its increasing frequency, much remains unclear regarding its pathophysiology. These uncertainties, and areas mandating further study, will be delineated.

## Diagnosis and epidemiology of congenital hypothyroidism

The introduction of newborn screening for CH has been a major public health success, enabling early commencement of levothyroxine in affected infants and thereby significantly ameliorating CH-associated physical and neurodevelopmental morbidity. Following the detection of elevated thyroid-stimulating hormone (TSH) or subnormal thyroxine (T4) levels (depending on the regional neonatal screening protocol), the diagnosis of CH is substantiated by venous thyroid function tests. A European consensus has determined that a raised TSH level of greater than 20 mU/L and free T4 (FT4) concentration below the age-appropriate reference range confirm a diagnosis of primary CH and mandate prompt treatment with levothyroxine (3). Thyroid imaging may also be helpful in differentiating between thyroid dysgenesis and GIS CH/dyshormonogenesis, thereby providing limited prognostic information (2).

Forty years ago, the majority of CH cases were attributed to thyroid dysgenesis. Over time, the incidence of CH has increased worldwide; key recent epidemiological studies are summarized in Table 1. Many CH cohorts now show a larger overall proportion of cases exhibiting GIS CH with a stable incidence of thyroid dysgenesis (2, 4, 5) although thyroid ectopy had increased in one study (6).

**Table 1:** Summary of key studies evaluating the epidemiology of CH over time.

Reference	Time period	Country	CH incidence	TCH incidence	Likely causes
Chiesa *et al.* (13)	1997–2010	Argentina	GIS CH increased in incidence	No significant change	Change in TSH cut points. (partly). Possible contribution of iodine deficiency
Kara et al (7)	2008–2010	Turkey	Two-fold increase in PCH, five-fold increase in TCH since past evaluations with higher TSH cut point (20 mU/L)	52% cases had TCH. TCH increased from 35% (2008) to 56% (2009–2010) when TSH cut point further decreased	Increased incidence of PCH and TCH partly due to decreased TSH cut points; High overall incidence of TCH possibly due to I- deficiency
Mitrovic *et al.* (6)	1983–2013	Serbia	Overall CH incidence tripled as TSH cut point decreased. PCH due to ectopy/GIS/goitre doubled, athyreosis stable.	TCH increased from 0 to 35%	Decreased TSH cutoffs; Other yet unidentified factors.
Mitchell *et al.* (9)	1991–1994; 2001–2004	USA	CH incidence doubled due to increased cases with delayed or mild TSH elevation. Severe CH stable.	TCH stable	Mainly due to enhanced detection of infants with mild disease and premature infants with delayed TSH rise due to altered screening strategy
McGrath *et al.* (14)	1979–2016	Ireland	Increased incidence of CH from 0.27 (1979–1991) to 0.65 cases per 1000 live births (treated 2005–2016). Mainly mild CH with normal or hyperplastic GIS.	TCH only assessed in final study period	Not due to change in TSH cut points or population ethnicity. Environmental factors, for example, iodine insufficiency, may have contributed
Hinton *et al.* (12)	Summary of total and state-specific data 1991–2007	USA	CH incidence increased in the United States by 3% per year; however, an increase did not occur in all states, at a constant rate, or at the same rate	Not assessed	Race, ethnicity, sex, and low birth weight/preterm birth all affected CH incidence.
Albert *et al.* (11)	1993–2010	New Zealand	Overall incidence of CH rose from 2.6 to 3.6 per 10 000 live births due to increased GIS CH.	Not assessed	Mainly due to altered population ethnicity. No change in TSH cut points.
Harris & Pass (10)	1978–2005	USA	Overall incidence of CH rose 73% between 1987 and 2002	Not assessed	Altered demographics account for 36–38% of the increase in incidence of CH. Diagnostic cut points unchanged.
Deladoey *et al.* (8)	1990–2009	Canada	Incidence of GIS CH doubled, and TD and goitre remained constant.	Not assessed	Decrease in TSH cut pojnt
Barry *et al.* (5)	1982–2012	France	Annual average increase of 5.1% for GIS, mild and severe. TD constant.	Not assessed	Unlikely due to change in TSH cut points or population ethnicity. Possible contribution of increased preterm birth and iatrogenic iodine overload

PCH, permanent CH; TD, thyroid dysgenesis.

In some regions, the increased incidence of CH is largely due to increased detection following the lowering of screening thresholds or altered screening algorithms (6, 7, 8, 9). However, factors underpinning the increased incidence are variable and may include changes in the ethnic mix of the screened populations (10, 11, 12), as well as increasing preterm birth and survival rates due to advances in perinatal medicine (12). More speculative reasons may include environmental contributors such as iodine deficiency and other as yet unidentified factors (7, 10, 13, 14, 15).

A smaller number of studies have specifically evaluated the proportion of CH attributable to TCH, with most demonstrating an increase over time, such that this may now include more than one-third of children with GIS CH (6, 7, 16). However, some studies have shown stable incidence of TCH (9, 13). Studies designed to evaluate the effect of decreased TSH screening cutpoints on the incidence of CH have frequently found a predominant increase in mild permanent GIS CH, presumably due to mild dyshormonogeneses which may also be unmasked by environmental determinants, when screening cutpoints are decreased (4, 17). However, others have found that the diagnosis of TCH is increased with lower TSH cut points (18).

## Physiological thyroid hormone biosynthesis requirements and implications for CH

Circulating thyroid hormone concentrations in euthyroid individuals are at their highest in the neonatal period but decrease rapidly during infancy, reflecting a significant decline in thyroid hormone requirements during the first year of life (19). Subsequently, further, transient increases in thyroid hormone biosynthesis requirements are observed during puberty and throughout pregnancy (in females) (20). Exogenous levothyroxine requirements reflect these altered metabolic demands, and therefore, medication doses require frequent adjustment in CH, particularly in the first few weeks and months of therapy, aiming to restore TSH into the normal range and to maintain an FT4 concentration in the upper half of the normal age-related reference range. FT4 concentrations above the upper limit of the reference interval can be accepted if the TSH is within the age-specific reference range (3). Initial levothyroxine requirements are usually 8–15 μg/kg/day and levothyroxine should be commenced promptly following diagnosis of CH. In infants with negligible thyroid function (e.g. those with CH due to ectopy or athyreosis), the change in levothyroxine dose requirements with age is similar and therefore predictable (21).

**Figure 1:**
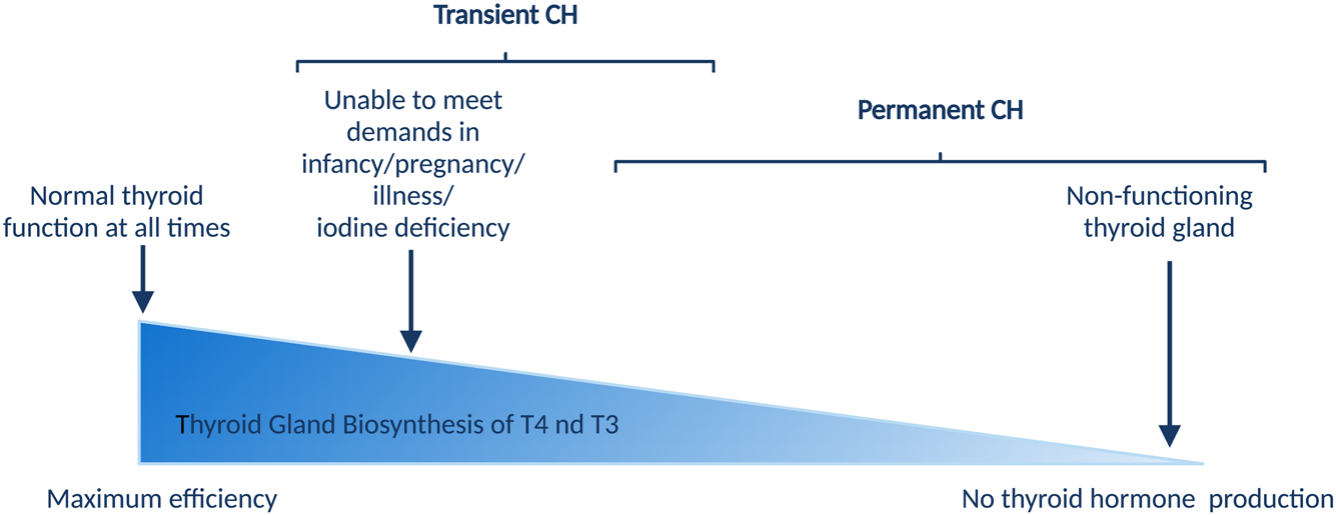
Association between the efficiency of thyroid hormone biosynthesis and transient CH. Created with BioRender.com.

However, GIS CH includes a spectrum of variably severe hypothyroidism, in which the magnitude of thyroid dysfunction will be indirectly proportional to the efficiency of thyroid hormone biosynthesis (Fig. 1). Some individuals with only partially impaired thyroid hormone biosynthesis due to specific endogenous defects, for which alternative pathways can functionally compensate, may be able to maintain euthyroidism at times when thyroid hormone requirements are at basal levels although levothyroxine supplementation may be required at times of increased metabolic demand, for example, during the neonatal period and times of rapid growth. Consequently, when thyroid hormone biosynthesis declines in early childhood, levothyroxine treatment can be withdrawn in these individuals, resulting in a diagnosis of TCH. In other individuals, the transiency of hypothyroidism reflects transient exposure to an environmental precipitant.

## Establishing a diagnosis of transient congenital hypothyroidism

Unfortunately, it is not usually possible to distinguish individuals with permanent CH from those in whom CH will be transient at the time of diagnosis, although several studies have evaluated predictors of CH duration. The best indicators of TCH seem to be lower levothyroxine requirements throughout the course of treatment, lower TSH levels at diagnosis in comparison to those seen in permanent CH, and absence of TSH elevation during treatment (2, 22, 23, 24). Additional predisposing factors noted in some studies include low birth weight, male sex, non-White ethnicity, prematurity and neonatal intensive care admission, and maternal thyroid disease (22, 25, 26). However, given the difficulties in predicting TCH, all children with GIS CH should be reassessed before the end of the third year of life to determine whether a trial without levothyroxine treatment is warranted.

Based on the evidence to date, the suggested indications for treatment cessation include the absence of a definitive diagnosis of permanent CH, a daily dose of thyroxine ≤ 25 μg (consider if <3 μg/kg/day) and stable or decreasing dose requirements, without TSH rise on treatment (3). The most recent European consensus advises that levothyroxine should be stopped or weaned over 4–6 weeks with re-evaluation of thyroid function 4 weeks after treatment cessation. Euthyroid biochemistry according to an age-appropriate reference range suggests that the child has TCH. If the FT4 levels remain within the reference range and the TSH measurement is either within the reference range or marginally elevated (less than 10 mU/L), re-evaluation with a repeat thyroid function test is recommended after a further 4 weeks of treatment (3).

Children with TCH should not necessarily be assumed to have had ‘mild CH’ during infancy as the degree of thyroid hormone deficiency in the neonatal period may be profound, with complete deficiency of FT4 and significantly elevated TSH concentrations. The severity of neonatal hypothyroidism will dependent on the underlying aetiology, which may be genetic, environmental or a combination of both (27).

## Neonatal hyperthyrotropinaemia

The developing fetus begins to produce TSH from 12 weeks with a steady increase in concentrations until adult values are reached by term (28). This is likely to represent the maturation of the hypothalamo–pituitary axis with a corresponding increase in T3 and T4 concentrations over this time period. Postnatally, there is a TSH surge within minutes and hours of birth and which returns to baseline in the first days of life (29) and an associated peak in T4 concentrations (30). Primary TSH screening programmes use cut points that take the timing of this TSH surge into consideration and are designed to minimize false-positive referrals. The UK cut point is set at 8 mU/L taken on days 5–10.

Neonatal hyperthyrotropinaemia with elevated TSH and normal FT4 concentrations is a distinct category of transient thyroid dysfunction which remains poorly understood. It may represent a delay in the resolution of the postnatal TSH surge, persistence of relative fetal pituitary insensitivity to thyroid hormone (28), and/or in some cases, there may be aetiological overlap with CH. Sequential biochemistry demonstrates the normalization of thyroid function over time without a requirement for treatment.

If FT4 becomes subnormal or the TSH exceeds 20 mU/L, the child is considered to have CH and levothyroxine treatment is required. The long-term implications of raised TSH in the neonatal period are not understood, but there is a suggestion of a relationship between TSH concentrations and poorer neurodevelopmental outcomes (31, 32). A proportion of affected individuals have also been found to have subclinical hypothyroidism in later childhood (32).

## Preterm infant and thyroid dysfunction

Prematurity is associated with a characteristic thyroid dysfunction with a blunted TSH surge and a delay in the timing and magnitude of serum T4 and T3 concentrations that correlate with gestational age (30). The pathophysiology underlying this ‘hypothyroxinaemia of prematurity’, is multifactorial, and in most preterm babies, serum T4 levels gradually rise and match those seen in term babies by 4–8 weeks of age.

However, an increased proportion of preterm babies, in particular those with very low or extremely low birth weight, exhibit CH characterized by delayed TSH elevation following an unremarkable first newborn screening test (30). Such cases usually have low/borderline low T4 necessitating levothyroxine treatment and imaging studies often show a normal GIS. Few studies have evaluated the outcome of CH in these infants (33).

Although studies are likely to be limited by other confounders associated with preterm birth, prematurity has been reported as an independent risk factor for CH. Studies evaluating large CH cohorts demonstrate enrichment of preterm cases (4, 16, 34). However, the data are conflicted in defining whether prematurity is associated with permanent (17) or transient dysfunction (35, 36) or both (37), and the extent to which preterm birth can be used as a reliable predictor of TCH is not clear. Barry* et al* (15) confirmed a significant association but others have generally failed to implicate gestational age in this role (22, 24, 38). TCH in preterm babies may be consistent with the recognized vulnerability of the immature thyroid to environmental causes of transient dysfunction including suboptimal or excessive iodine status (18, 30, 36).

## Understanding the pathophysiology of transient CH

**Figure 2:**
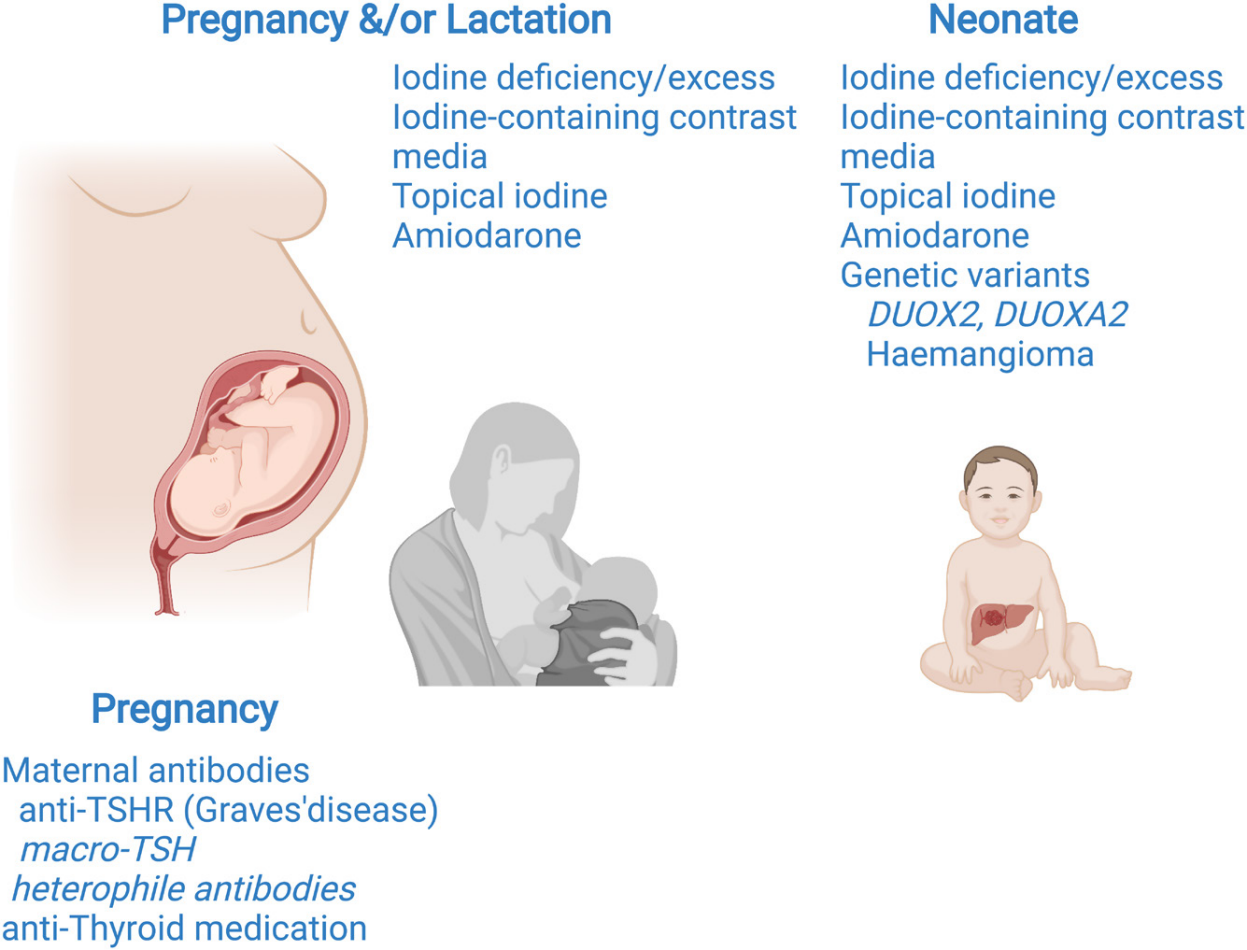
Summary of causes of transient CH which may involve maternal environment during gestation, nutritional composition and passage of medication in breast milk, and neonatal exposure to unfavourable iodine status, contributory medication, and the presence of specific genetic mutations or haemangioma. Artefactual causes are shown in italics. Created with BioRender.com.

Unfortunately, there are no large cohort studies investigating the pathophysiology of TCH, one of the main limitations for prospective studies is the need for CH cases to undergo re-evaluation at the age of 3 years, in order to establish the transiency of thyroid dysfunction. Therefore, most evidence supporting the determinants of TCH is based on anecdotal reports of well-characterized cases or small case series. Both genetic and environmental causes have been implicated in the pathophysiology of TCH and most exert their influence on the thyroid hormone biosynthesis pathway (Fig. 2).

## Thyroid hormone biosynthesis

**Figure 3:**
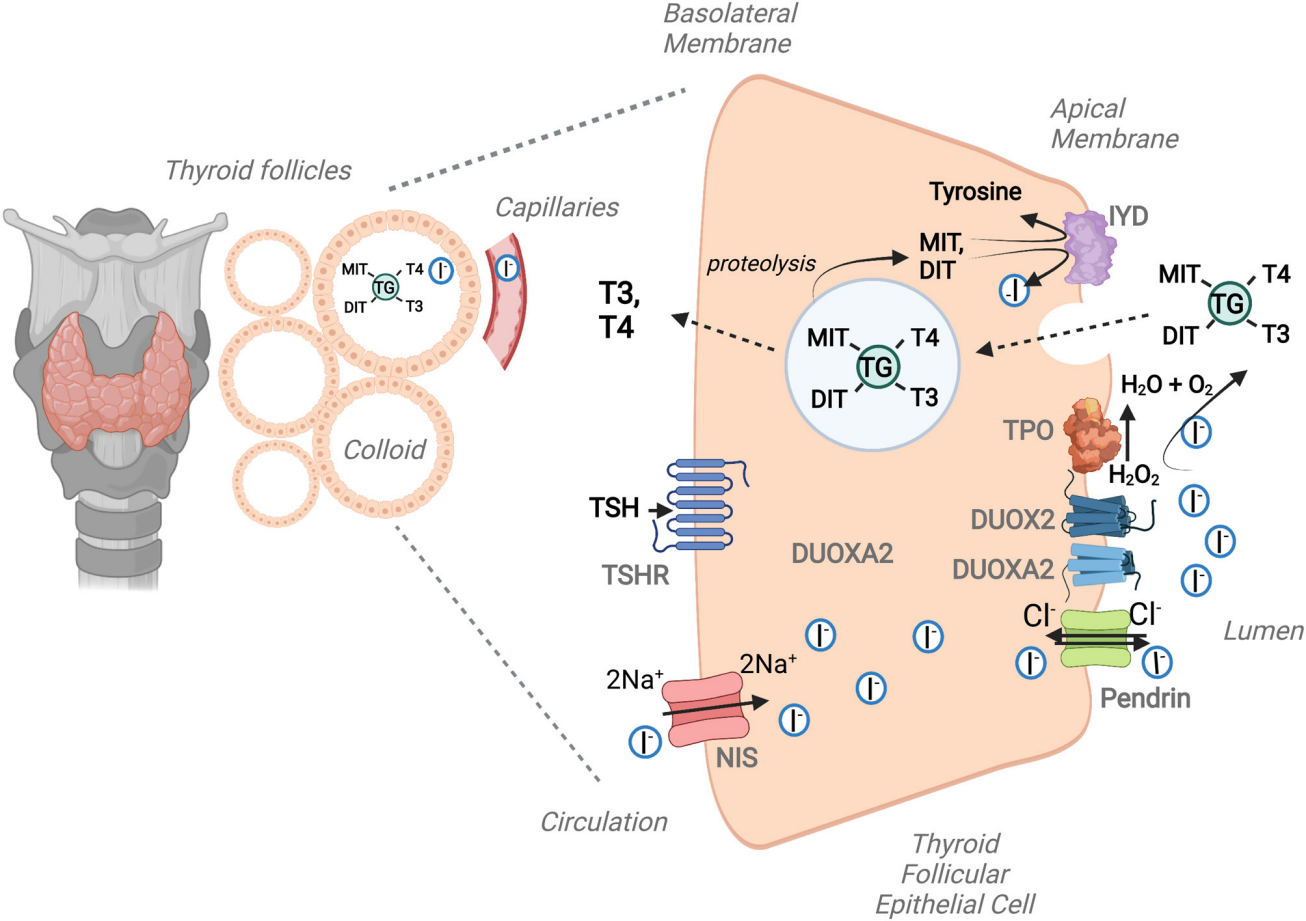
Schematic illustrating the key molecules required for thyroid hormone biosynthesis. Mutations in any of these molecules (TSHR, NIS, Pendrin, TG, TPO, DUOX2, DUOXA2, IYD) may cause CH and mutations in the NADPH-oxidase DUOX2 and its accessory protein DUOXA2 are particularly implicated in transient CH. Created with BioRender.com.

Thyroid hormone biosynthesis is mediated by transporter molecules and enzymes expressed by thyroid follicular cells and requires adequate circulating iodide substrate and thyroglobulin (TG) for iodination (Fig. 3). Causes of TCH may include inappropriate supplies of iodide substrate or defective genes encoding the molecules responsible for incorporating iodide into thyroid hormones or recycling unused mono and di-iodotyrosyl (MIT and DIT).

During normal thyroid hormone biosynthesis, iodide is taken up from the circulation across the basolateral membrane of the thyrocyte by the sodium-iodide symporter (NIS, SLC5A5) which coordinates the electrogenic symport of two sodium ions for one iodide ion, down an electrochemical gradient generated by the Na^+^/K^+^ ATPase. Specific transporters, for example, pendrin (SLC26A4) and anoctamin-1 then mediate iodide efflux into the follicular lumen, where it is oxidized in the presence of hydrogen peroxide (H_2_O_2_) and incorporated into tyrosyl residues on the surface of TG to form MIT and DIT. MIT and DIT couple to form thyroid hormones (T4 and triiodothyronine (T3) which are endocytosed back into the thyroid follicular cell and then cleaved and secreted into the circulation.

The thyroid peroxidase enzyme, TPO, catalyzes the H_2_O_2_-dependent oxidation of I^−^ into I^+^, the iodination of tyrosyl residues, and the coupling of MIT and DIT. DUOX2 (an NADPH-oxidase enzyme) and its accessory protein, DUOXA2, are the predominant source of H_2_O_2_. Monocarboxylate transporter 8 (MCT8) and other transporters mediate TH efflux from the thyroid gland and iodotyrosine deiodinase (IYD) recycles unused iodide moieties (1). The anion transporter SLC26A7 has also been shown to play a key role in thyroid hormonogenesis although its molecular role in the thyroid has not yet been characterized (39, 40). Mutations in any of the key molecules involved in thyroid hormone biosynthesis may cause CH (Fig. 3).

## Genetic causes of transient CH

Several studies from different countries have robustly implicated* DUOX2* and *DUOXA2* in the aetiology of transient CH (27, 41, 42). DUOX2 is an NADPH-oxidase enzyme required for hydrogen peroxide biosynthesis in the thyroid and its maturation factor DUOXA2 is required for DUOX2 trafficking from the endoplasmic reticulum to the plasma membrane. Both proteins are eventually expressed at the apical membrane in close proximity to TPO and continue to have direct functional interactions with each other. There is potential functional redundancy in the thyroidal DUOX system due to concomitant expression of *DUOX1*, which is contiguous with *DUOX2* on the long arm of chromosome 15 and encodes an additional thyroidal NADPH-oxidase. The respective DUOX1 and DUOX2 maturation factor genes, *DUOXA1* and *DUOXA2,* occupy the *DUOX* intergenic region. DUOX1 and DUOX2 proteins exhibit 83% sequence homology; however, DUOX2 is thought to be the dominant isoenzyme in the thyroid, as evidenced by its higher thyroidal expression levels, and observations that, in humans, monogenic mutations in both *DUOX2* and *DUOXA2*, but not *DUOX1* or* DUOXA1*, have been implicated in CH.

*DUOX2* mutations are frequently reported in individuals with CH, especially those of East Asian ethnicity, with more than 120 disease-causing and 110 likely disease-causing mutations listed in HGMD Professional 2021.2. Furthermore, an analysis of known CH-associated genes in the gnoMAD population database estimates that *DUOX2* has the highest carrier frequency of pathogenic variants (43). *DUOXA2* mutations occur less frequently with only 23 disease-causing and 14 likely disease-causing mutations reported in HGMD Professional 2021.2.

Intriguingly, despite clear enrichment of DUOX2 and DUOXA2 variants in cohorts with CH, the occurrence of pathogenic variants in these genes in an apparently healthy population (e.g. gnoMAD) is more frequent than compatible with the prevalence of GIS CH. The true association of DUOX mutations with CH may be underestimated due to false-negative newborn screening results in mutation carriers if conventional (higher) TSH screening cut points are used or if the TSH rise is delayed (27). However, it is possible that *DUOX2* and *DUOXA2* mutations may strongly predispose to CH in the setting of particular genetic or environmental modulators but are not causative in isolation.

Unlike in humans, DUOX2 loss of function is associated with severe, permanent CH in murine models and is also thought to mediate the permanent hypothyroidism and slow metabolic rate seen in giant pandas (44, 45, 46). To the best of our knowledge, there are no known rodent genetic models of TCH, which limits the investigation of the underlying mechanisms for human TCH. Additionally, in murine models, only *DUOX2,* and not *DUOX1*, loss of function is associated with hypothyroidism; thus, the role of DUOX1 in thyroid biology remains unclear (46). However, it has been speculated that, in humans, DUOX1 upregulation in the context of DUOX2 loss of function may at least partially compensate for defective H_2_O_2_ production, thus explaining the frequent transiency of DUOX2-associated CH. Inadequate compensatory H_2_O_2_ production by DUOX1 may result in CH in the neonatal period, when thyroid hormone biosynthesis is at its peak, or perhaps when there is a particular temporal requirement for DUOX2 activity which later declines. However, when thyroid hormone biosynthesis requirements decline during later childhood, DUOX1 and/or other H_2_O_2-_producing enzymes may sufficiently compensate for the DUOX2 deficit to maintain euthyroidism, resulting in the resolution of CH (47).

In support of this notion, more than 50% of individuals with CH associated with *DUOX2* or *DUOXA2* mutations exhibit TCH, including those harbouring biallelic-truncating *DUOX2* mutations, which abolish the H_2_O_2_-generating domains and presumably abrogate DUOX2 activity completely (27, 47). In contrast, a report describing digenic, homozygous *DUOX2* and *DUOX1* mutations in one family notes uncharacteristically severe CH in the affected siblings, consistent with perturbed compensatory mechanisms for H_2_O_2_ production when both DUOX enzymes are disrupted (48).

Studies have yielded variable results regarding the association between the number of *DUOX2* mutations and permanency or transiency of CH, but in general, the proportion of TCH and phenotypic severity seem similar with both biallelic and monoallelic mutations and are unrelated to the site or nature of the mutation (27, 47, 49). One study reported that CH associated with one or two *DUOX2* mutations was more likely to be subclinical or transient, whereas CH in the context of three or more *DUOX2* mutations was more likely to be permanent (50). However, *DUOX2* mutations are associated with variable penetrance and poor genotype–phenotype correlation, suggesting heterogeneity in the efficiency of any compensatory processes, likely due to either genetic or environmental modulators (47). Both *DUOX2* and *DUOXA2* mutations may be associated with borderline TSH elevation on neonatal screening followed by a delayed TSH rise and profoundly subnormal FT4 level, resulting in CH which is severe, albeit transient (27).

Defects involving other genes involved either in thyroid hormone biosynthesis pathway or thyroid development usually cause permanent CH, even if mild. This may reflect the fact that in contrast to DUOX2, there are no alternative pathways to compensate for the specific defect in other mild dyshormonogeneses, for example, due to TG or TPO mutations. Transiency in these contexts would therefore require a second reversible ‘hit’, for example, iodine deficiency temporarily unmasking a phenotype. Although compensated hypothyroidism may occur with TSHR mutations, this represents a permanent compensated state, in which upregulation of TSH synthesis and a possible ‘resetting’ of the hypothalamic–pituitary–thyroid axis maintains TSH elevation despite normal pituitary sensitivity to circulating thyroid hormones (51). A single case of TCH has been reported in association with a heterozygous missense mutation in *PAX8*, one of the key thyroid transcription factors which is essential both for thyroid morphogenesis and adult thyroid architecture and function (52).

## Iodine status

Iodine is an essential trace mineral substrate required for thyroid hormone biosynthesis and both iodine insufficiency and excess may cause TCH.

## Iodine deficiency

Iodine deficiency causes a spectrum of adverse effects resulting from compromised thyroid hormone production due to inadequate iodine substrate, with endemic cretinism representing the most severe manifestation of iodine deficiency *in utero*. Although the number of countries with adequate iodide intake has almost doubled over the last 20 years due to salt iodization, 21 countries worldwide remain iodine-deficient (53). The neonatal thyroid is much more susceptible to iodine deficiency than the adult thyroid since thyroidal iodide content at birth is extremely low and daily neonatal iodide turnover (17% increasing to 125% in severe iodine deficiency) is accelerated compared with adults (daily iodide turnover 1%). Maternal iodine deficiency may result in compensatory neonatal TSH elevation, with associated thyroglobulin elevation and increased thyroid volume, depending on the severity of the deficit (54).

Historically, several lines of evidence support a causal role of iodine deficiency in the pathogenesis of TCH. Shortly after the introduction of newborn screening for CH, TCH was found to be almost eight times more prevalent in Europe, where iodine deficiency was common, compared with North America, where iodination of salt maintained iodine sufficiency (55). Further examples of frequent occurrence of TCH in areas of mild-moderate iodine deficiency have been reported worldwide; in Algeria, the incidence of CH (predominantly transient) varied from 0.22% in a severely iodine-deficient region to 0.09% in iodine-replete region (56). Additionally, in Bursa, a moderately iodine-deficient region of Turkey, a particularly high incidence of TCH was reported (total incidence of CH, 1/840 and permanent CH, 1/2354) (57). More recently, a systematic review and meta-analysis investigating the association of neonatal TSH with maternal iodine status during pregnancy and the early postpartum period found that TSH levels in cord samples of neonates born to mothers with iodine deficiency were significantly higher than those with maternal iodine sufficiency (58).

The role of iodine deficiency in the aetiology of TCH has also been confirmed by observations that iodine supplementation is preventative, such as in studies supplementing Belgian preterm infants with 30 μg potassium iodide/day (55). Additionally, in iodine-deficient area of Poland, initiation of an iodine supplementation programme ameliorated the incidence of TCH (59). Although severe iodine deficiency is associated with profound hypothyroidism, especially in areas such as Zaire, where thiocyanate overload synergizes with iodine deficiency to cause thyroid failure, the thyroid dysfunction may also be transient and is preventable by maternal iodine supplementation before or during pregnancy (55).

It has been speculated that iodine deficiency may partly explain the recent increases in incidence of TCH, observed in several population studies (4, 7, 14), especially since iodine deficiency has been demonstrated to be more common than previously anticipated, including during pregnancy, even in countries with strong healthcare systems (60). Re-emergence of iodine deficiency has recently been reported in the UK and Australia despite previous surveys suggesting the populations were iodine-replete (61, 62). National Health and Nutrition Examination Survey (NHANES) data also demonstrate a deterioration in iodine status of pregnant women in the United States from marginally iodine-sufficient in 2001–2006 to mildly iodine-deficient in 2007–2010 (63, 64).

However, the degree to which declining iodine status contributes to increasing incidence of TCH has not been evaluated in detail, and in some cohorts, iodine deficiency is thought not to be implicated (11). Moreover, recent studies comparing maternal iodine status and neonatal TSH levels in mild-moderately iodine-deficient populations have yielded heterogenous results, perhaps due to additional pre- or postnatal factors or the sample size and timing of measurements. Therefore, although a strong contender for mediating some of the observed increase in TCH, the relative contribution of iodine deficiency to contemporary TCH, alone or in concert with genetic and/or other environmental factors remains unclear in most areas (65, 66).

## Iodine excess

Excess iodine can also result in hypothyroidism by interfering with thyroid hormone biosynthesis through the Wolff–Chaikoff effect. This autoregulatory effect was first described in 1948 and is still incompletely understood. Thyroid peroxidase activity is inhibited, which may be partially explained by increases in iodolactone, iodoaldehyde and/or iodolipid levels, and hydrolysis of thyroglobulin may also be impaired (67, 68).

Usually, a normally functioning thyroid gland ‘escapes’ from the Wolff–Chaikoff effect after around 2 weeks by downregulation of the sodium–iodide symporter, NIS such that the subsequent decrease in intracellular iodide concentration permits renewed synthesis of thyroid hormones (69). However, the fetal thyroid gland cannot escape from the Wolf–Chaikoff effect until around 36 weeks gestation; therefore, both fetal and neonatal thyroid (especially in preterm infants) are very susceptible to iodine excess, from 18 to 20 weeks, when fetal thyroidal iodide uptake begins (68). In the fetus, iodine can be absorbed through the skin or gastrointestinal tract from amniotic fluid or acquired by placental transfer. Since iodine is excreted through breastmilk, maternal iodine intake during lactation will influence neonatal iodine status, and low neonatal renal iodine clearance, following active neonatal thyroidal iodide trapping can result in blockade of thyroidal iodine transport for weeks to months by iodine excess (70).

## Excess iodine consumption

### Food products or supplements

Iodine overconsumption can occur through food products, predominantly those containing seaweed, which is central to some East Asian cultures, for example, in Japan and Korea. In this setting, excessive iodine intake has been associated with subclinical hypothyroidism in preterm infants in Korea, where women traditionally consume iodine-rich brown seaweed soups postpartum (71, 72). Maternal consumption of seaweed during pregnancy and during lactation has also anecdotally been associated with neonatal transient or mild persistent CH, both in Asian and non-Asian families (70, 73). Maternal intentional or inadvertent consumption of iodine supplements (e.g. in herbal remedies) may also result in TCH and maternal consumption of inorganic iodine (4–100 mg/day) for thyrotoxicosis, while breastfeeding has been associated with mild TCH in 10% infants (74, 75, 76).

### Topical iodine

Topical iodine-containing disinfection agents have been implicated in TCH, both when directly involving the neonate or when used by the mother during pregnancy, labour, or lactation (77, 78). This is particularly relevant in preterm infants where the effects of even brief exposure can result in marked hypothyroidism (79, 80). In Iran, the use of povidone–iodine during delivery resulted in an increased recall rate during CH screening and a higher median TSH, compared with a group in whom chlorhexidine was used instead. Historically, evaluation of an Italian TCH cohort with a high percentage of preterm infants identified iodine exposure in more than 50% of cases (81). The use of iodine as a disinfectant in the peri- and neonatal period is now not recommended, due to the risk of TCH (3, 82).

### Iodine-containing contrast

Iodine contrast used during pregnancy or in very early infancy may also cause TCH. During pregnancy, since currently used iodinated contrast media (ICM) are water-soluble and readily cleared from the body, fetal exposure to high iodide loads is transient. High osmolar, lipid-soluble ICM exhibit delayed excretion and confer a greater risk of hypothyroidism. A systematic review of neonatal thyroid function following the use of ICM before or during pregnancy found a tendency towards an increased risk of thyroid dysfunction with higher doses of contrast, especially when injected into the fetal compartment during amniofetography (83). However, small studies suggest that maternal administration of water-soluble ICM (e.g. iodohexol) during pregnancy does not usually cause CH (84, 85). Thyroid function in neonates conceived after hysterosalpingography with ICM also seems generally not to be affected (86).

Neonatal administration of ICM may also cause hypothyroidism, especially in premature neonates; in a systematic review, 8.2% of term infants and 18.3% of premature infants developed hypothyroidism after ICM exposure (87, 88). Individuals with congenital heart disease are particularly vulnerable as they may be exposed to multiple sources of excess iodine simultaneously, including large intravenous contrast loads during cardiac catheterization and topical iodine-containing antiseptics and dressings following surgical procedures. One study revealed a diagnosis of CH in 25% neonates with congenital heart disease following iodine exposure during these procedures and a four-fold increase in CH in neonates undergoing more than three procedures (88, 89).

## Drugs

### Amiodarone

The commonly used anti-arrhythmic agent amiodarone comprises 37% iodine and shares structural similarities with T4. The high iodine load may provoke hypothyroidism by the Wolf–Chaikoff effect, and since the significant transplacental passage of amiodarone and its derivative desethylamiodarone occur, both neonatal and *in utero* exposure may cause neonatal TCH. Amiodarone is used for both maternal and fetal dysrhythmias, and over 20% of neonates exposed to amiodarone *in utero* exhibit hypothyroidism which may be biochemically profound and is sometimes associated with goitre; hypothyroidism may develop after only a few weeks of fetal exposure (90). Amiodarone-induced hypothyroidism due to administration in the neonatal period may also occur, although fewer case reports describe neonatal exposure compared with ⋘ fetal exposure (91).

### Maternal antithyroid drugs (ATDs)

Poorly controlled maternal hyperthyroidism during pregnancy is associated with adverse outcomes for the fetus, and mother, and therefore requires treatment with thionamide medication. Propylthiouracil (PTU) is used most commonly as it carries the lowest risk of teratogenicity; however, later in pregnancy, methimazole (MMI), or its prodrug carbimazole, is sometimes used. Thionamide medication can cross the placenta, and since the kinetics of placental transfer are similar for PTU and MMI, they each carry a similar risk of causing fetal hypothyroidism or neonatal TCH (92, 93). Some studies have shown poor correlations between maternal antithyroid drug dose and fetal thyroid status, and cord serum PTU concentrations may also be higher than simultaneously obtained maternal serum PTU concentrations, suggesting slower PTU clearance in the fetus (94). Therefore, although there is a correlation between maternal and fetal T4 levels, fetal thyroid status may be suppressed even though maternal thyroid status seems normal on thionamide treatment .

Generally, clinically evident fetal hypothyroidism is rare at low doses of PTU (≤50 mg daily) and a recent study showed no significant differences between maternal and cord FT4 levels at this dose (95). Both methimazole and PTU are rapidly cleared from the fetal circulation; therefore, neonatal-transient hypothyroidism due to ATDs usually resolves within a few days and may not trigger the newborn diagnostic screening programme (96).

## Antibody-mediated transient CH

### Maternal transfer of antibodies

Maternal autoimmune hyperthyroidism may impact fetal thyroid status through the pathogenesis of the disease itself, in addition to the effects of antithyroid medication. Graves’ disease is associated with the development of autoantibodies targeting the TSH receptor (TRAb, anti-TSHR antibodies) which may be stimulating, blocking, or bio-inactive in nature. Different anti-TSHR antibody subtypes can coexist and can cross the placenta to the fetus from mid-gestation onwards (96, 97). A high titre of blocking antibodies can lead to profound hypothyroidism in the newborn and failure of uptake of technetium during thyroid imaging, which may lead to a presumptive diagnosis of athyreosis if thyroid ultrasound or thyroglobulin measurement is not undertaken (98). Depending on the titre of antibodies, these may take 3–6 months to clear the circulation following which hypothyroidism will resolve in most cases. However, it is reported that potent-blocking antibodies may also impair development of the thyroid *in utero* leading to permanent CH (99).

The clinician should become suspicious of the diagnosis when the pattern of levothyroxine dose requirements and thyroid biochemistry becomes inconsistent with athyreosis. A careful history and measurement of maternal thyroid function and antibody status may assist with early identification of this cause of TCH such that unnecessary prolonged treatment can be avoided and appropriate monitoring is undertaken in future pregnancies due to risk of recurrence (100).

### Immunoglobulin interference in TSH assays (artefactual TCH)

**Figure 4:**
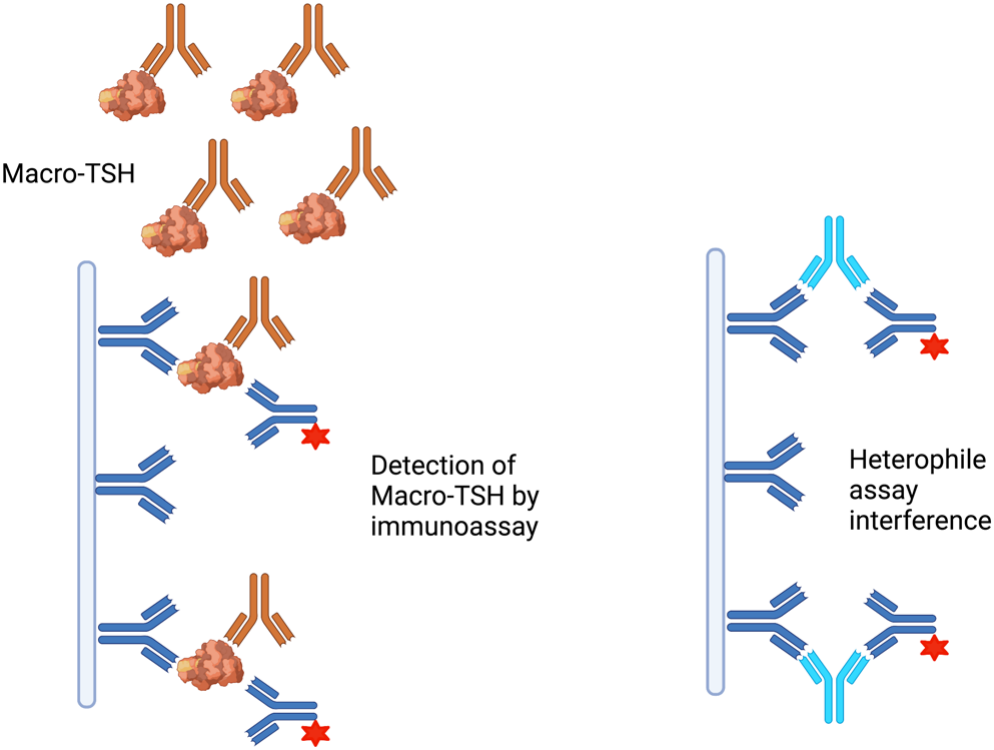
Schematic demonstrating TSH assay interference by heterophile antibodies (targeting assay reagents) and macro-TSH, which refers to the presence of circulating, bioinactive TSH held in complex by immunoglobulins. Although bioinactive, macro-TSH is immunoreactive and may be detected by laboratory immunoassays, resulting in spuriously high readings. Both types of antibody may affect fetal TSH results by transplacental passage. Created with BioRender.com.

Artefactual TCH may occur due to transplacental passage of maternal antibodies which interfere with the TSH assay, causing spuriously high TSH results. Although FT4 and FT3 are usually normal in this context, significantly high TSH levels may result in misdiagnosis of CH and inappropriate levothyroxine treatment. This phenomenon may occur both with anti-animal or heterophile (poly-specific) antibodies targeting assay reagents and with anti-TSH antibodies resulting in the formation of macro-TSH complexes (Fig. 4). TSH measurements for affected individuals may be grossly discrepant between different assays due to variable effects of interfering antibodies in the different assay platforms and the recovery of TSH may be non-linear to dilution. Polyethylene glycol (PEG) precipitation to remove high molecular weight proteins results in low recovery of TSH; although method-dependent, and when PEG-precipitable TSH exceeds 90% (i.e. recovery of TSH is <10%), clinicians should strongly suspect the presence of macro-TSH (101). The presence of macro-TSH can be confirmed using gel filtration chromatography to demonstrate a high molecular weight TSH peak fraction that approximates the molecular size of IgG and to demonstrate the increased recovery of TSH following incubation of the macro-TSH sample in serum from a patient with primary hypothyroidism (102). Macro-TSH has an estimated prevalence of 0.79% in patients with subclinical hypothyroidism (TSH > 4 mIU/L) (103).

Transplacental passage of maternal-interfering immunoglobulins usually causes elevated TSH with normal FT4 and FT3 in a clinically euthyroid neonate, and a similar pattern of thyroid biochemistry is identified in the mother (104, 105). It is therefore crucial to measure maternal thyroid function in this context, as an elevated maternal TSH with normal FT4 and FT3 supports the diagnosis, and confirmation of artefactually elevated TSH will avoid inappropriate levothyroxine treatment in a clinically euthyroid baby or inappropriate genetic investigations for a presumed autosomal dominant condition. Interference in the TSH assay resolves around 8 months of age in accordance with a normal rate of elimination of maternal IgG, whereas the TSH level of the mother remains high.

### Haemangioma

Infantile haemangiomas (IH) are the most common tumours of infancy, occurring in up to 10% of infants, and they follow a characteristic pattern of proliferative growth during the first year of life, followed by gradual involution. Diffuse infantile hepatic haemangiomas are frequently associated with consumptive hypothyroidism, due to high levels of type 3 iodothyronine deiodinase (D3) expression. D3 is a selenoenzyme, normally present in brain, placenta, and fetal liver and catalyses the conversion of T4 to reverse T3 (rT3) and the conversion of T3 to 3,3’-diiodothyronine, both of which are biologically inactive. In consumptive hypothyroidism, elevated TSH is associated with normal free T3 (fT3), normal or low-free T4 (fT4), and elevated serum rT3 levels as a result of increased T4 and T3 degradation by D3 (106, 107). Extrahepatic haemangiomas have also been associated with consumptive CH (108). Hypothyroidism usually resolves in parallel with regression of the hepatic lesions but requires treatment with escalating doses of levothyroxine, with or without liothyronine, in addition to medical management of the haemangiomas themselves, which may include the administration of propranolol, vincristine, interferon, and corticosteroids (27, 109).

## Future directions

TCH is increasing in incidence and, despite recognized genetic and environmental causes, there are no large-scale cohort studies investigating its basis and the reasons for this increase. Smaller studies have suggested the involvement of hitherto uncharacterized factors (110). Consequently, the relative contributions of the known aetiologies remain speculative and likely differ between geographical cohorts. Historically, iodine exposure played a prominent role in TCH, but given the consequent recommendations against neonatal iodine exposure, and the use of alternative agents (e.g. chlorhexidine) where possible, it is unlikely that this is driving the contemporary increased incidence. Maternal thyroid disease is also unlikely to be a major player since its incidence is likely to have remained stable and blocking TSHR antibodies is rare (81).

The most convincing potential contributors identified so far include the effects of increased ascertainment due to decreased screening cut points, the likely re-emergence of iodine deficiency in certain regions, and altered genetic background or ethnicity of the screened population, which may have changed over time. In some settings, increased survival of preterm infants may also contribute.

All of these factors may interact; for example, DUOX-associated TCH may only be detected if screening cut points are low, iodine deficiency may unmask hypothyroidism in the setting of a mild dyshormonogenesis, and the preterm thyroid may be more vulnerable to genetic or environmental insult. However, there is a clear need for comprehensive studies evaluating genetic, environmental, and demographic factors in large TCH cohorts, in order to clarify the relative roles of these factors alone and in combination.

Additionally, the long-term outcomes of children with TCH require evaluation, especially in regard to whether reintroduction of levothyroxine at time points of increased metabolic demand and growth may be required in children with underlying genetically mediated dyshormonogenesis. This may be most relevant during pregnancy where fetal development and pregnancy outcomes are dependent on euthyroid status.

Finally, it remains disputed whether TCH always requires treatment, and it is sometimes not categorized as ‘true CH’ in epidemiological studies (111). Since TCH may be profound, there are strong grounds for detection and treatment at the severe end of the biochemical spectrum. However, further studies are required to address the neurodevelopmental implications of mild TCH and the benefits of treatment, especially where the duration of the deficit is brief.

## Conclusion

TCH is increasing in incidence, and although there are several well-defined genetic and environmental determinants, further studies are required to evaluate their relative contributions. Children with TCH will usually have a normally located thyroid gland *in situ* and may have lower than predicted thyroid hormone requirements. However, TCH cannot be predicted on the basis of initial neonatal screening biochemistry or thyroid scintigraphy. Moreover, although the association of TCH with *DUOX2* and *DUOXA2* mutations is well-recognized, CH duration cannot be predicted on the basis of genotype alone. Clinicians should assess whether a child with CH is likely to have permanent or transient CH by 3 years of age or earlier and trial individuals off levothyroxine therapy as indicated. When TCH is confirmed to be genetically mediated, the individual should be offered long-term monitoring for recurrent thyroid dysfunction, particularly during pregnancy.

## Declaration of interest

The authors declare that there is no conflict of interest that could be perceived as prejudicing the impartiality of this review.

## Funding

N S is funded by the Wellcome Trust (Grant number: 219496/Z/19/Z) and supported by the NIHR Cambridge Biomedical Research Centre. The views expressed are those of the authors and not necessarily those of the NIHR or the Department of Health and Social Care.

This research was funded in part, by the Wellcome Trust (Grant numbers 219496/Z/19/Z). For the purpose of open access, the authors have applied a CC-BY public copyright licence to any Author Accepted Manuscript version arising from this submission in accordance with the grant’s open-access conditions.
